# Accuracy of Rhythm Diagnostic Systems’ MultiSense^®^ in Detection of Arterial Oxygen Saturation and Respiratory Rate During Hypoxia in Humans: Effects of Skin Color and Device Localization

**DOI:** 10.3390/s25010127

**Published:** 2024-12-28

**Authors:** Charles Evrard, Amina El Attaoui, Cristina Pistea, Irina Enache, Mark Marriott, Louis Mayaud, Anne Charloux, Bernard Geny

**Affiliations:** 1 Biomedicine Research Center of Strasbourg (CRBS), UR 3072, “Mitochondria, Oxidative Stress and Muscle Plasticity”, Faculty of Medicine, University of Strasbourg, 67000 Strasbourg, France; evrard.charles@outlook.com (C.E.); cristina.pistea@yahoo.fr (C.P.); irina.enache@chru-strasbourg.fr (I.E.); anne.charloux@chru-strasbourg.fr (A.C.); 2 Department of Physiology and Functional Explorations, University Hospital of Strasbourg, 67000 Strasbourg, France; 3 Rhythm Diagnostic Systems SAS, 67000 Strasbourg, France; amina.elattaoui@rdsdiag.com (A.E.A.); mark.marriott@rdsdiag.com (M.M.); 4 Institut Hospitalo-Universitaire, 67000 Strasbourg, France

**Keywords:** hypoxia, sensors, pulse oximeter, pulse oxygen saturation, SpO_2_, respiratory rate, wearable device

## Abstract

The continuous monitoring of oxygen saturation (SpO_2_) and respiratory rates (RRs) are major clinical issues in many cardio-respiratory diseases and have been of tremendous importance during the COVID-19 pandemic. The early detection of hypoxemia was crucial since it precedes significant complications, and SpO_2_ follow-up allowed early hospital discharge in patients needing oxygen therapy. Nevertheless, fingertip devices showed some practical limitations. In this study, we investigated the reliability of the new Multisense^®^ pulse oximetry system compared to a reference pulse oximeter (Vyntus CPX Pulse Oximeter) during hypoxia. In a population of sixteen healthy male subjects (mean age: 31.5 ± 7.0 years, BMI: 24.9 ± 3.6 kg/m², and 35% with darker skin tones), simultaneous SpO_2_ and RR measurements were collected over 12.4 h, during which FiO_2_ was progressively reduced from 21% to 10.5%. The average root mean square error (ARMS) of SpO_2_ for Multisense^®^ placed on the back and chest was 2.94% and 2.98%, respectively, with permutation testing confirming a significant ARMS below 3.5% for both positions and no statistically significant difference in the ARMS between patch placements. Positive correlations and acceptable accuracy between devices were observed at both locations (r = 0.92, *p* < 0.001 and r = 0.90, *p* < 0.001 for back and chest placements, respectively). Bland–Altman analysis further indicated limits of agreement that support consistency across placements, with similar agreement levels noted across skin tones. Similar findings were obtained with the RR measurements. In conclusion, Multisense^®^ demonstrated robust accuracy in measuring SpO_2_ and RRs during hypoxia in humans comparable to standard hospital-grade equipment. The effectiveness of the findings suggests that this wearable device is a valuable tool for the continuous monitoring of SpO_2_ and RRs, potentially enhancing patient safety and optimizing hospital resource allocation. Nevertheless, to overcome study limitations and allow generalized use, further work on a larger population sample, including more subjects with a high phototype and desaturation below 80%, would be useful.

## 1. Introduction

Many clinical situations are associated with impaired oxygenation leading to a vicious circle where hypoxemia (low blood oxygen levels) enhances ischemia deleterious effects (impaired perfusion), thereby decreasing the lung and heart capacities to capture and deliver oxygen to tissues already showing signs of hypoxia. Thus, in addition to numerous pathologies resulting in acute or chronic respiratory failure (asthma, chronic obstructive pulmonary disease (COPD), interstitial pneumopathy, pulmonary viral or bacterial infection, etc.), heart failure, whatever its causes (ischemic cardiopathy, systemic hypertension, and valvular diseases), also results in acute or chronic lung edema, inducing systemic hypoxia. Physiological situations might also be associated with hypoxia. Thus, walking at high altitudes can be life-threatening involving pulmonary edema and hypoxic vasoconstriction due to decreased oxygen saturation [1,2].

Hypoxemia can be silent, and even the major symptoms, dyspnea and respiratory rates (RRs), can vary, making it difficult to manage the patient’s health efficiently [3,4,5,6,7]. Hypoxia awareness might also need to be developed in aircrew members [8]. These data support the need and interest of monitoring a physiological parameter that might allow early diagnosis of a pathology’s aggravation and a continuous non-invasive and easy-to-perform follow-up of a patient’s oxygenation. Accordingly, telemedicine, in general, and continuous monitoring of oxygen saturation (SpO_2_), in particular, became a major issue in many cardio-respiratory diseases. Indeed, they allow at-home follow-up and can prevent hospitalization without reducing the patient’s prognosis [9,10,11,12,13]. Such monitoring was also shown to be of tremendous importance during the COVID-19 pandemic [14,15]. Particularly, the occurrence of hypoxemia needed to be detected early, since it preceded rapid and significant complications. Further, SpO_2_ follow-up likely allowed early hospital discharge in patients needing oxygen therapy and those who did not [16]. Monitoring tissue oxygenation is also very interesting to assess acclimatization to high altitudes and follow athletes using hypoxia to ameliorate their cardiorespiratory and muscular responses to heavy and/or prolonged exercise [17,18,19]. In this view, a better definition and knowledge of liability should reduce doctors, caregivers, and patients’ reluctance to use sensors and artificial intelligence in medicine, allowing wider use and thus fulfilling the promise of sensors to improve patient care [20].

A pulse oximeter is a device that measures the oxygen saturation of arterial blood non-invasively [21]. In general, pulse oximeters use two-wavelength absorption spectrophotometry to measure oxygen saturation. The wavelengths are selected to provide the best separation of absorbances of oxy-hemoglobin and deoxy-hemoglobin states. The ratio of the two absorbances allows the oxygen saturation (SpO_2_) value to be calculated. As an arterial sample of blood is not required to make the measurement, the pulse oximeter can provide continuous, non-invasive, real-time information. The clinical use of pulse oximeters has reduced the frequency and necessity of invasive arterial blood sampling and has helped to improve subject safety by providing continuous information to clinicians about subjects’ oxygenation status [22].

Nevertheless, with the widespread use of fingertip pulse oximeters, these devices have notable practical limitations. This study, therefore, aimed to evaluate the reliability of the novel Multisense^®^ pulse oximetry system in comparison with a hospital reference pulse oximeter under controlled hypoxia conditions. Additionally, we assessed the potential influence of device placement on SpO_2_ measurement accuracy. The MultiSense^®^ solution is a wearable medical device developed by RDS SAS (Strasbourg, France) intended for continuous remote monitoring of clinical parameters and designed to enhance patient management and quality of life. This adhesive patch has built-in sensors that provide wireless and non-invasive measurements of blood oxygen saturation (SpO_2_) and respiratory rates. Considering its relevance, hypoxia is commonly associated with alterations, including pulmonary hypertension, which is very frequent in humans suffering from sleep disorders, e.g., obstructive sleep apnea, during which recurrent apneas or hypopneas result in intermittent hypoxemia with systemic inflammation and cardiovascular complications. Therefore, we rigorously tested the Multisense^®^ system’s (Vyaire Medical GmbH, Hoechberg, Germany) performance in detecting hypoxemia.

The primary objective of this study was to evaluate the accuracy and reliability of the innovative Multisense^®^ pulse oximetry system (device under test: DUT) during controlled hypoxia in healthy volunteers in comparison with a reference pulse oximetry system (Vyntus CPX Pulse Oximeter standard of care: SOC (Xpod^R^ SpO_2_)). Additionally, we examined the impact of skin pigmentation, using the Fitzpatrick scale and Munsell Dermatology Rating (MDR) [23,24,25,26], on the accuracy of SpO_2_ and respiratory rate measurements by the Multisense^®^ device. To evaluate potential variations in data quality, we compared SpO_2_ and respiratory rate measurements from a patch worn on the anterior chest and a patch worn on the upper back.

## 2. Population and Methods

### 2.1. Population

The inclusion criteria of the population participating in the study were men between 18 to 50 years of age, the ability to receive and understand information related to the study and give written informed consent, and non-smokers or ex-smokers (stopped smoking for at least 6 months). At the level of the population, the subject demographics included a range of dark skin pigmentations, including at least 30% of the total pool, assessed by phototypes V and VI according to the Fitzpatrick scale (a questionnaire score between 28 and 40). Further, the volunteers needed to be healthy, as assessed by physical examination, a normal ECG, and the absence of medical treatment.

The exclusion criteria included the antecedent of allergies to adhesives or silicone or a skin disease that would preclude the use of an adhesive, implantable device, such as a pacemaker, a high-altitude stay during the previous 4 weeks (more than 1 week spent over 3500 m), and Raynaud’s syndrome.

All volunteers gave their informed consent before participating in the study. The clinical trial was conducted in compliance with the Declaration of Helsinki, and the national ethics committee approved the study (CPP Ile de France 11, 21-048-34245, obtained on the first of August 2021 and registered on ClinicalTrials.gov: NCT05044585).

### 2.2. Study Design

This was a prospective, monocentric study. Adult healthy volunteers recruited in the respiratory functional exploration unit of Strasbourg’s NHC were equipped with two MultiSense^®^ devices (one on the chest and one on the back). To determine the quality of the measurements of arterial blood saturation (SpO_2_) provided by the MultiSense^®^ system, we compared the SpO_2_ values obtained simultaneously with a reference pulse oximetry system (Vyntus CPX Pulse Oximeter) and the MultiSense^®^ system in terms of accuracy. Accuracy was defined as an average measurement variation less than 3.5% of the mean value to the reference standard (ISO 80601-2-61, FDA, 2013) [27].

#### 2.2.1. Study Time Frame

The Clinical Investigation Center performed the subject recruitment and the Physiology and Functional Explorations Service teams performed the hypoxia procedure at the University Hospital of Strasbourg.

Prior to the screening visit, the volunteering subjects received the information notice and the informed consent form for the study by mail.

The first screening visit was conducted two days before the experiment. It allowed written informed consent, the collection of relevant medical history and demographic data (sex, date of birth, height, weight, and the color of skin by a Melanin Densimeter measurement and the FitzPatrick questionnaire) to be obtained. Physical examinations and ECG 12 leads were also performed.

The second visit was the day of the hypoxia test. We applied the two data acquisition patches for assessment, one on the sternum and one on the upper back (see the monitoring procedure below) before starting data acquisition with the MultiSense^®^ patches and applied the reference monitor.

#### 2.2.2. Investigational Device and Reference Monitor Descriptions

##### General Description of the Investigational Device

**Figure 1:**
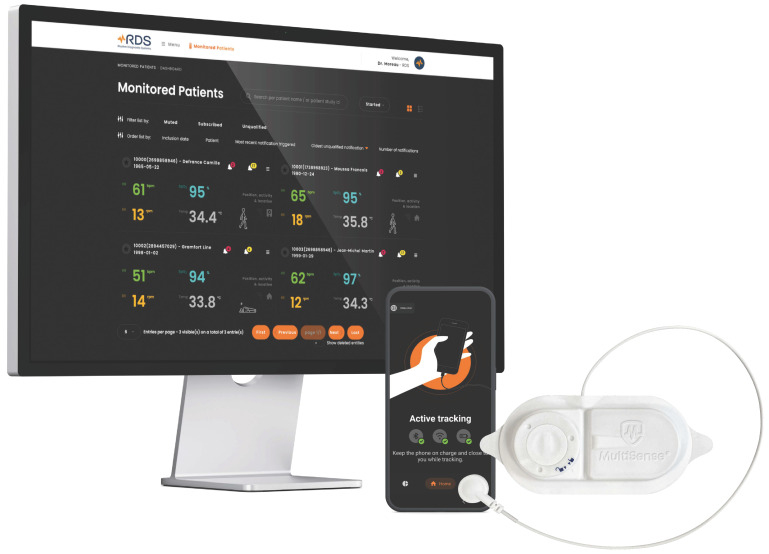
Overall architecture of RDS Multisense solution.

MultiSense^®^ is a solution for the remote measurement of clinical data designed to facilitate patient monitoring and improve patient quality of life in non-critical care settings (Figure 1). It is manufactured by RDS SAS (1 Place de l’hôpital, 67000 Strasbourg, France) and allows medical professionals to remotely and continuously monitor their patients’ clinical parameters.

It is composed of four main parts:

- An innovative multiparametric device in the form of a single miniaturized adhesive patch that is placed on the upper body to collect physiological data continuously;
- A phone, connected by Bluetooth to the patch, equipped with an application that receives the data collected and sends it to the cloud using a wireless connection (cellular network or Wi-Fi);
- A GDPR and ISO27000-compliant cloud infrastructure for data storage and interpretation, running proprietary algorithms that derivate clinical parameters from the raw data collected by the patch;
- A secured web portal for medical professionals that allows them to access recent and historical data for their patients.

##### General Description of the Reference Monitor

We used the Vyntus CPX Pulse Oximeter (Vyntus CPX V-178001, Vyaire Medical GmbH, Leibnizstrasse7, 97204 Hoechberg, Germany) as the reference monitor. It consists of two components: an external wired PULSE Oximeter (based on NONIN Xpod^R^ SpO_2_) and an HR sensor (CE- and FDA-cleared). The Xpod^®^ 3012LP was developed to connect to a VO2 interface of the CPX and is described by the manufacturer as an “accurate, low-power, easy-to-integrate external pulse oximetry solution for medical products. Nonin PureSAT^®^ signal processing and PureLight^®^ sensor technologies combine for a highly responsive and accurate SpO_2_ monitoring system”. More characteristics are available at https://www.nonin.com/products/xpod/, (accessed on 20 December 2024).

#### 2.2.3. Description of the Hypoxia Test

**Figure 2:**
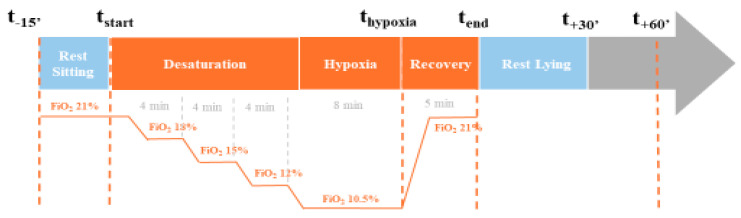
Kinetics of the hypoxia test.

Hypoxia tests were performed in the physiology and functional exploration unit. The flow of the protocol is summarized in Figure 2. At the beginning of the test, the patient was at rest, sitting in the study chair for 15 min prior to the start of the hypoxia condition (t-15′). Then, the hypoxia program started with a progressive shift from ambient air (FiO_2_ of 21%) to control gas flow using the AltiTrainer Isocap (GANSHORN Medizin Electronic GmbH, Niederlauer, Germany) device (Sport and Medical Technologies SA). The program consisted of decreasing the FiO_2_ by steps of 3%, holding 4 min plateaus with stabilized FiO_2_ from 21% (before desaturation started) to 18% (first plateau), 15% (second plateau), and 12% (third plateau). The last plateau was performed for 8 min at FiO_2_ of 10.5%.

At this step, the objective of the subject’s SpO_2_ was to be between 70% and 80%. After this acquisition, the subjects were given normal air to return them to baseline levels. Once the subjects had reached their initial saturation, the breathing circuit was disconnected.

### 2.3. Data Collection, Pre-Processing, and Quality Assessment

#### 2.3.1. Data Collection

SpO_2_ data were collected on a second-by-second basis from healthy volunteers using the MultiSense^®^ device placed at two locations (on the chest and on the back). A standard of care device (Vyntus CPX Pulse Oximeter) was used as the reference for comparison. The oxygen saturation levels of the subjects were experimentally controlled to range from 100% to 70%, simulating both desaturation and re-saturation phases.

#### 2.3.2. Data Pre-Processing

The time series data from the DUT and the SoC were aligned using the heart rate time series from both the DUT and the SoC. The time series were aligned using a temporal correlation analysis with a sliding window of one second going 2 min before and two minutes after the original time. A time offset, calculated based on the heart rate data, was applied to synchronize the SpO_2_ time series from the DUT with the SoC reference.

To ensure consistency and reliability, data were pre-processed by restricting the analysis to a specific time window. The start of the analysis was defined as 10 min before the first point at which the SoC SpO_2_ dropped below 89%; the analysis continued through the desaturation period to the minimum value recorded on the SoC. This method ensured that the most critical time periods for evaluating SpO_2_ accuracy were captured in the analysis, balancing all deciles of SpO_2_ values.

Additionally, any data points flagged as low quality by any device sensors were excluded from the analysis. Each sensor provided a confidence measure for its readings, and data points with low confidence were removed to ensure the analysis was based on high-quality data.

#### 2.3.3. Assessment of the Quality of Data Acquisition

The quantification of missing data was computed as the time-wise ratio of missing versus total signals. In addition, missing data were also characterized by the number of missing episodes (n) as well as measures of the spread and central tendency of the missing episode lengths. Stretches of artifactual data within the patch signal were identified using an algorithm specialized in detecting outliers in time series data. The percentage of the time series data recognized as being artifactual using this algorithm was summarized and reported. Offset and correlation were calculated as vectors containing, for any given measure, the patch and standard of care (SOC) time series data, respectively. In addition, the variance of patch and SOC were calculated and reported. Visualization is provided by way of Bland–Altman plots on a per-subject basis as well as across subjects.

### 2.4. Statistical Analysis

Analyses were performed using Python software with additional packages (version 3.9).

To assess whether the average root mean square error (ARMS) of oxygen saturation (SpO_2_) measurements from the device under test (DUT) placed on the chest and back differed significantly from a predefined threshold of 3.5, a permutation-based approach was applied. For each permutation, the SpO_2_ values throughout hypoxic conditions were shuffled in time, and the ARMS was calculated for each subject and device location between the permuted DUT SpO_2_ values and the standard of care (SoC) SpO_2_ values. For each permutation, the minimum (ARMS-3.5) of each patch location was added to a distribution under the null hypothesis (i.e., no temporal correlation between the SOC and the DUT data). The observed ARMSs for the back and chest placement were then compared to that distribution. A *p*-value below 0.05 indicated that the DUT’s ARMS was significantly lower than 3.5, demonstrating acceptable accuracy compared to the SoC.

The second analysis evaluating whether the placement of the DUT on the chest or back resulted in greater accuracy was also tested using permutation analysis. The primary metric used for comparison was the average root mean square error (ARMS) between the DUT and the SoC. Permutation testing was conducted by randomly swapping data between the chest and back placements (under the null hypothesis that they are equivalent), and the ARMS difference between the two placements was computed for each permutation. The observed ARMS difference was compared to the distribution of permuted differences to compute a p-value. A *p*-value of a minimum below 0.025 indicated that the chest placement was statistically superior, while a *p*-value above 0.975 indicated that the back placement was superior. Values between 0.025 and 0.975 indicated no significant difference between the two placements.

For the third analysis, which investigated the association between skin color and measurement accuracy, the combined ARMS is defined as the mean of the ARMSs from both the chest and back placements used as the dependent variable. Two linear regression models were fitted between the Fitzpatrick and MDR skin color metrics and the combined ARMS. All variables, including the ARMS, Fitzpatrick, and MDR, were normalized using their respective means and standard deviations. For each permutation, the slopes of the regression models for Fitzpatrick and MDR were computed, and the maximum slope from either the Fitzpatrick or MDR metric was recorded for each iteration. The observed slope was then compared to the maximum distribution of slopes from the permutations to assess whether any observed associations between skin color and the ARMS were stronger than expected by chance. For all permutation analyses, 5000 permutations were chosen. Using the distribution of the minimum or maximum statistic for a permutation allowed correction for multiple hypothesis testing whenever applicable.

Finally, to evaluate the agreement between the experimental device and standard device SpO_2_, Bland–Altman graphical plots were generated, and linear regression fit, mean, and upper 95% and lower 95% limits of agreement were calculated, as previously reported [28,29].

## 3. Results

### 3.1. Population Characteristics

The data are presented as mean ± SEM. Seventeen male subjects, with a mean age of 31.5 years (±7.0), weight of 81.3 kg (±13.8), height of 180.5 cm (±6.8), and a BMI of 24.9 kg/m² (±3.6), were included in the study. Sixteen patients’ data were analyzed since one participant was excluded due to very noisy ECG MultiSense^®^ data. All subjects were healthy, as inferred from their clinical characteristics, presenting at rest with a normal heart rate of 70.7 bpm (±8.1), ambient air saturation of 98.7% (±1), systolic blood pressure of 132.5 mmHg (±12.3), and diastolic blood pressure 78.4 mmHg (±14.6).

**Figure 3:**
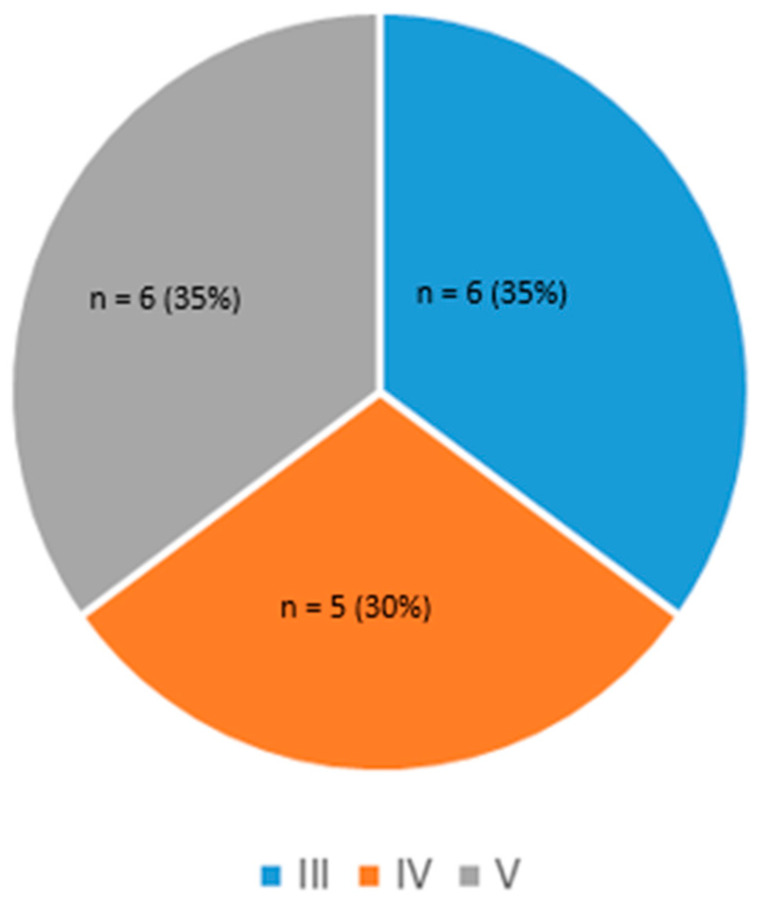
Skin phototype distribution.

An important metric for participant inclusion was a broad repartition of skin colors, with a minimum of 30% of the population from Fitzpatrick families V or VI. Accordingly, six participants out of 17 met this inclusion criterion (35%), as presented in Figure 3.

### 3.2. Comparisons Between the Two Devices

#### 3.2.1. Considering the Entire Population and Regardless of Skin Pigmentation and Device Positions

The primary objective of this study was to evaluate the accuracy and reliability of the MultiSense^®^ solution during controlled hypoxia in healthy volunteers. During the study, a total of 12.4 h of concomitant data collection was collected over 16 sessions and used for comparison analysis, with a mean length of 47 ± 3 min. Two parameters were analyzed: SpO_2_ and respiratory rate (RRs).

#### 3.2.2. SpO_2_ Measurements

##### ARMS Accuracy and Device Placements

**Figure 4:**
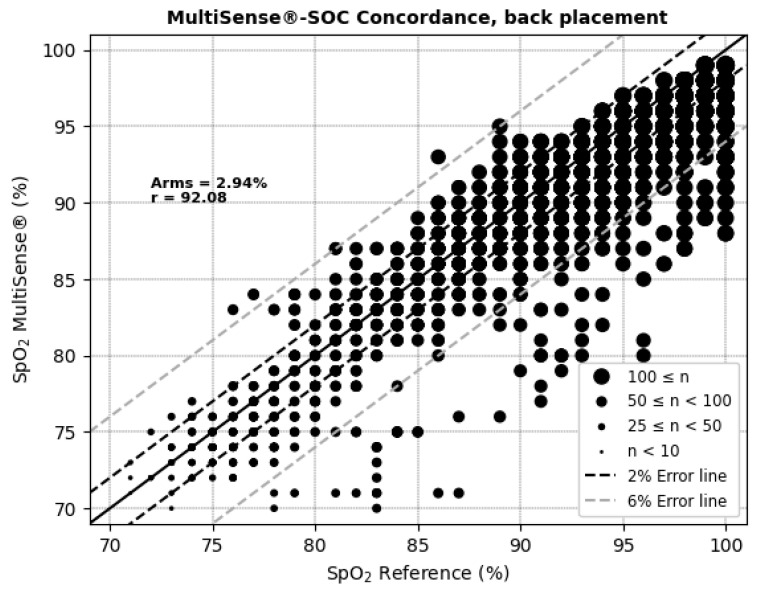
Concordance plot, ARMS (%), and Pearson correlation coefficient r (%) for SpO_2_, MultiSense^®^ in back placement.

**Figure 5:**
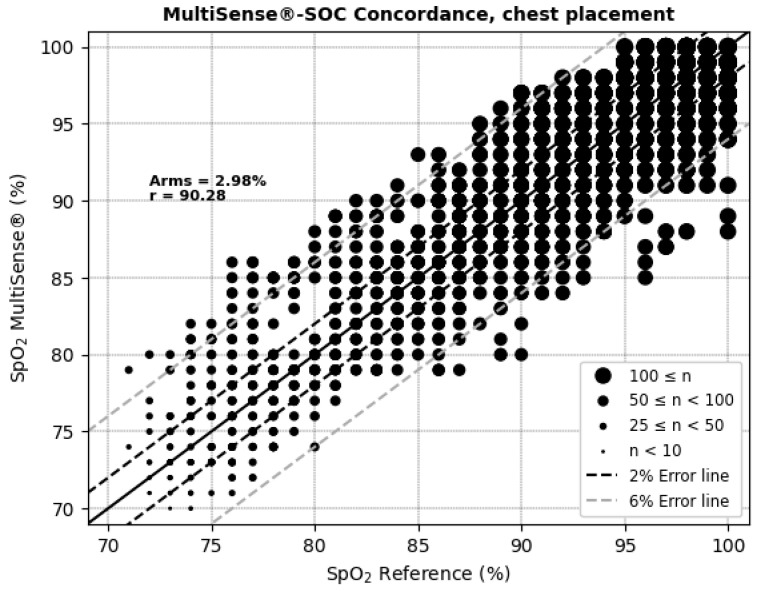
Concordance plot, ARMS (%), and Pearson correlation coefficient r (%) for SpO_2_, with MultiSense^®^ in chest placement.

The average root mean square error (ARMS) of SpO_2_ measurements for both the back and chest placements of the MultiSense^®^ device was 2.94% and 2.98%, respectively. The ARMS for the back placement was found to be significantly below 3.5% (*p* < 0.001), indicating that the device performed within acceptable accuracy limits for this location (Figure 4). Similarly, the ARMS for the chest placement was also significantly below 3.5% (*p* < 0.001), demonstrating comparable accuracy to the back placement (Figure 5). Additionally, the SpO_2_ values of both devices were highly positively and significantly correlated, whatever the device location (r = 0.92 and r = 0.90 in the back and chest placements, respectively). Correlation analysis measures the extent of the relationship between two variables; however, a strong correlation does not necessarily indicate good agreement between the two devices.

Therefore, a permutation test was conducted to assess whether there was a statistically significant difference in the ARMS between the chest and back placements. The *p*-value for the difference between the two placements was 0.52, indicating no significant difference in the accuracy of SpO_2_ measurements between the chest and back locations (*p* > 0.05). Thus, neither placement demonstrated a clear advantage over the other in terms of measurement accuracy.

**Figure 6:**
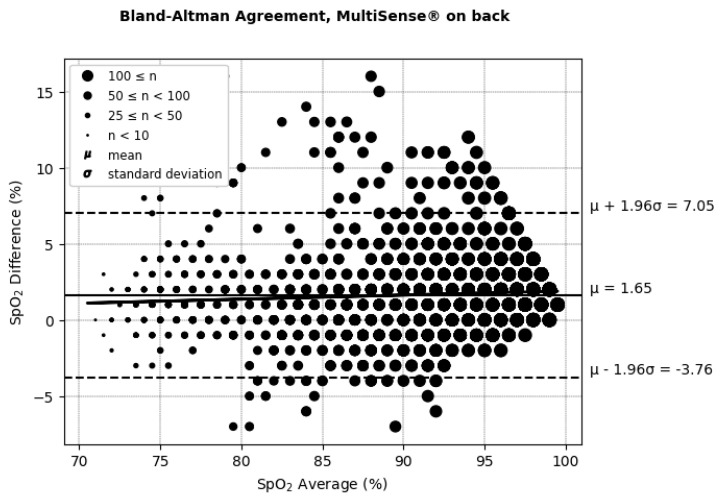
Bland–Altman limits of agreement for SpO_2_, MultiSense^®^ in back placement.

**Figure 7:**
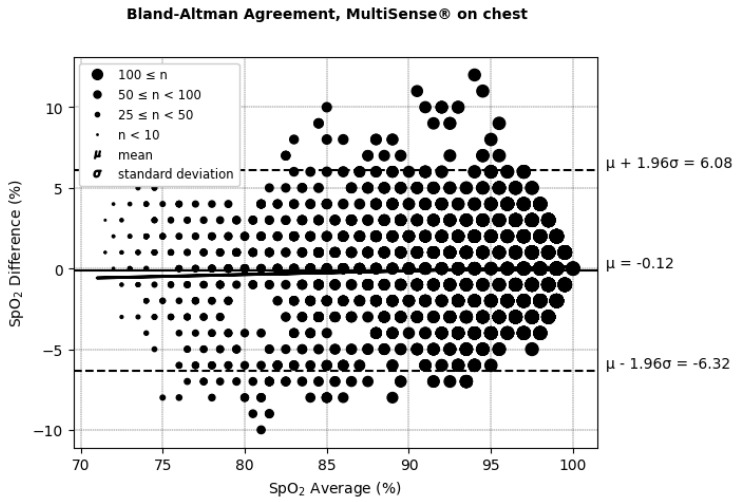
Bland–Altman limits of agreement for SpO_2_, MultiSense^®^ in chest placement.

The Bland–Altman plots (Figure 6 and Figure 7 and Table 1) depict the average SpO_2_ measurements from both the MultiSense^®^ and reference SoC along the x-axis, while the y-axis represents the difference in measurements between the two devices. For the back placement, we observed a mean bias of 1.65, with 95% of the differences falling within the limits of agreement, spanning from −3.76 to 7.05. For the chest placement, the mean bias was −0.12%, with limits of agreement between −6.32 and 6.08, encompassing 95% of the observed differences. These findings indicate that the SpO_2_ measurements from the MultiSense^®^ device align closely with the reference standard across both placements. Analysis plots reveal a distribution of deviations across the SpO_2_ range, with 18.77% (back) and 25.62% (chest) of points exceeding the ±3.5% difference threshold. For the back placement, deviations outside this range primarily appear in the mid-to-upper SpO_2_ levels, whereas for the chest placement, they are distributed similarly but with a slightly higher frequency. Despite these outliers, the majority of data points fall within clinically acceptable limits, suggesting that the MultiSense^®^ maintains robust agreement with the reference device. The observed spread of deviations does not cluster at extreme SpO_2_ values, indicating a stable performance across a wide operational range.

##### No Influence of Skin Color on ARMS

The overall SpO_2_ results were achieved by considering 35% of subjects with a high phototype, which exceeds the theoretical threshold of 30% required for SpO_2_ validation.

To investigate the potential influence of skin color on measurement accuracy, the relationship between the ARMS and two skin color metrics–the Fitzpatrick scale and the Munsell Dermatology Rating (MDR)–was analyzed using linear regression and permutation under the null hypothesis. For both the MDR and Fitzpatrick scale, no statistically significant associations were found with the ARMS. The *p*-value for the slope between the MDR and ARMS was 0.28, and for the Fitzpatrick scale, the *p*-value was 0.47, indicating that skin color, as assessed by these scales, did not have a statistically significant effect on the accuracy of the SpO_2_ measurements.

#### 3.2.3. Respiratory Rate Measurements

##### ARMS Accuracy and Device Placements

**Figure 8:**
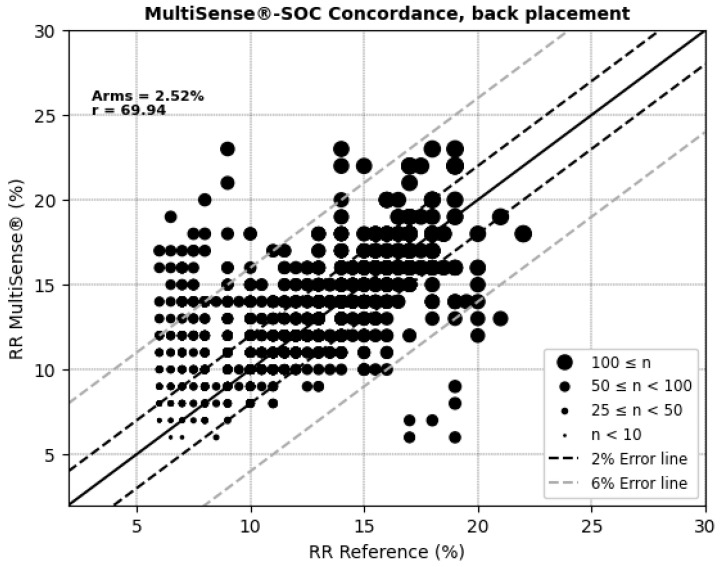
Concordance plot, ARMS (%), and Pearson correlation coefficient r (%) for RRs, MultiSense^®^ in back placement.

**Figure 9:**
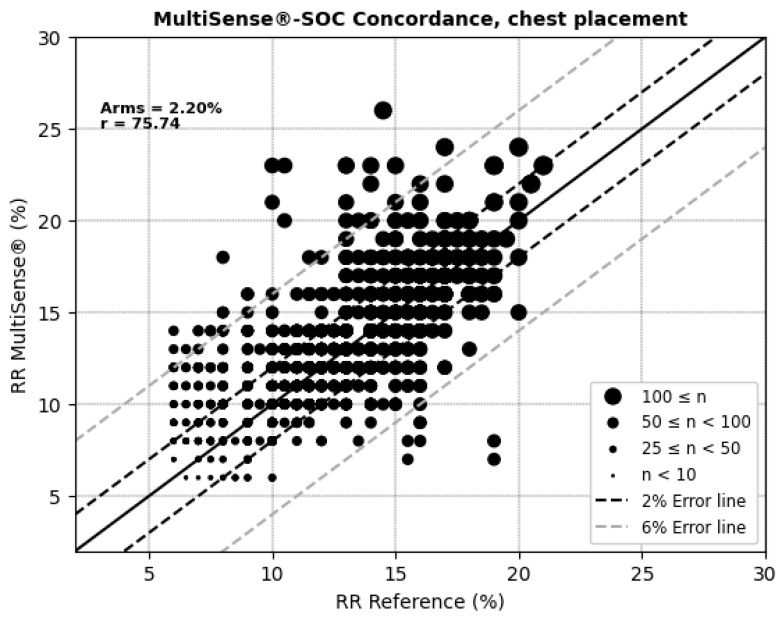
Concordance plot, ARMS (%), and Pearson correlation coefficient r (%) for RRs, MultiSense^®^ in chest placement.

For respiratory rate (RR) measurements, the MultiSense^®^ device achieved average root mean square errors (ARMSs) of 2.52% in the back position and 2.20% in the chest, both within clinically acceptable accuracy limits (Figure 8 and Figure 9). Additionally, significant positive correlations were observed with the reference device in both placements (r = 69.94% for the back and r = 75.74% for the chest).

A *p*-value of 0.42 was obtained from the permutation test, indicating no significant difference in the accuracy of the RR measurements between the chest and back locations (*p* > 0.05). Thus, neither patch placement demonstrated a clear advantage over the other in terms of measurement accuracy.

**Figure 10:**
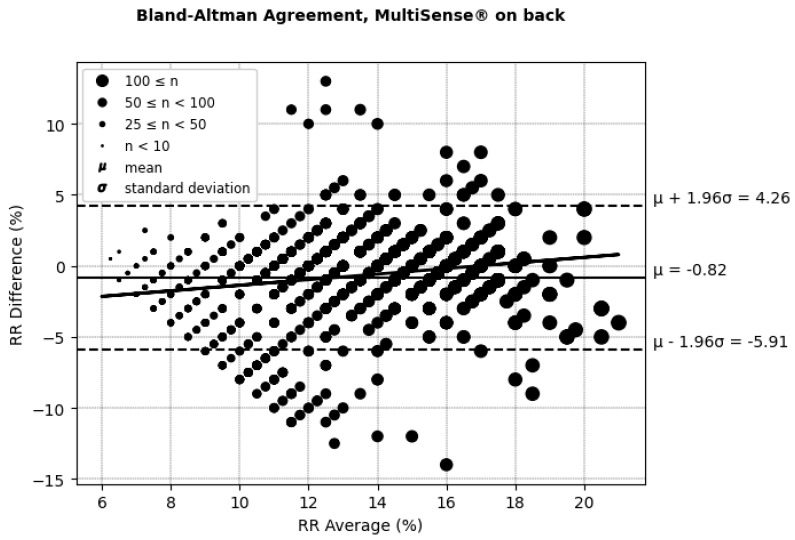
Bland–Altman limits of agreement for RRs, MultiSense^®^ in back placement.

**Figure 11:**
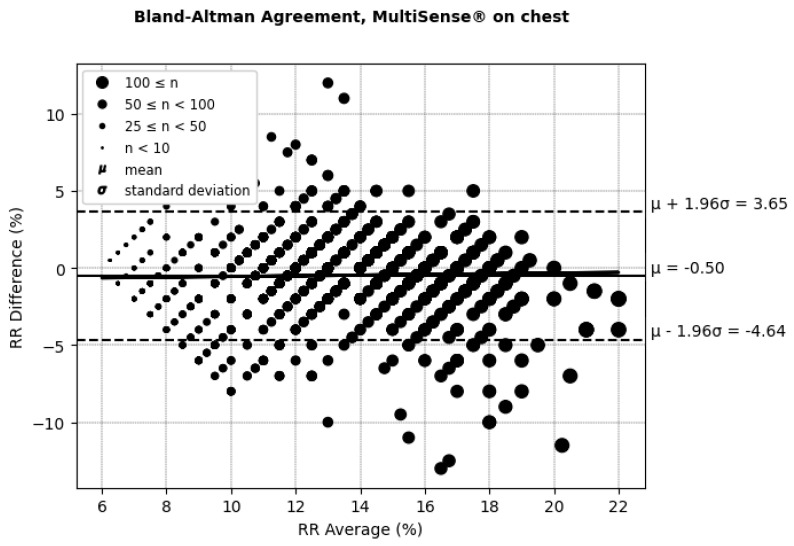
Bland–Altman limits of agreement for RRs, MultiSense^®^ in chest placement.

According to (AAMI, 2013) [30], the clinically acceptable boundaries for respiratory rate (RRs) often vary depending on the context and device requirements but are typically considered to be within ±5 breaths per minute or around ±10% of the reference measurement. These thresholds account for minor deviations that generally do not affect clinical decision-making. In the RR Bland–Altman analysis (Figure 10 and Figure 11 and Table 2), the MultiSense^®^ device demonstrated limits of agreement that align closely with these boundaries (−5.91 to +4.26 for the back placement and −4.64 to +3.65 for the chest placement), indicating that the observed deviations remain within an acceptable range for clinical applications.

Looking closer at the distribution, in the back placement, 14.64% of the points fall outside a ±5 rpm difference, while for the chest placement, only 9.34% of points exceed this threshold. Despite the presence of some outliers, most data points remain within clinically acceptable limits. These findings demonstrate that the MultiSense^®^ maintains clinically acceptable accuracy in measuring RRs, and no significant difference is obtained regarding patch placement. The distribution of points within the ±5 rpm range further supports the reliability of MultiSense^®^ in providing accurate respiratory rate measurements, highlighting its potential options in remote monitoring applications.

#### 3.2.4. Safety Assessment Considering Adverse Effects and Events

Eleven adverse events were recorded during the study. Eight participants experienced mild adverse events related to skin redness after device removal. Those skin reactions were limited to redness with no irritation and were due to the use of a solvent wipe to remove the adhesive patch after the study. They resolved spontaneously in the following hours.

Further, one participant presented with a neurocardiogenic syncope, and two experienced cardiac adverse events (an abnormal ECG and isolated extrasystoles). In all of these cases, the symptoms resolved spontaneously within the same day.

No serious adverse events were, therefore, observed.

## 4. Discussion

### 4.1. Effectiveness and Safety of the Device MultiSense for SpO_2_ and Respiratory Rate Measurements

The main findings of this study demonstrate that the MultiSense^®^ device provides accurate measurements of SpO_2_, achieving a comparable performance to the standard pulse oximetry device (Vyntus CPX Pulse Oximeter) when assessing oxygen saturation in humans under hypoxic conditions. The study’s main objective was to evaluate the reliability and clinical relevance of the innovative MultiSense^®^ pulse oximetry system, specifically during controlled hypoxia in healthy, stationary volunteers. This clinical trial is the first to assess the MultiSense^®^ device’s performance with the patch applied to the back, as the device was originally designed for chest placement.

In terms of accuracy, the comparison with the Vyntus CPX Pulse Oximeter yielded an ARMS of 2.94% for the back placement, which is statistically below the acceptability threshold of 3.5%. This result indicates that MultiSense^®^ accurately measures SpO_2_ levels during desaturation phases. Notably, such accuracy was observed in both the chest and back device placements and remained acceptable, even in the case of black skin color, which could not be statistically associated with the ARMS. The study design allowed for the proper representation of high phototypes (35%), which reinforces the quality of the results. These results are consistent with a recent publication showing that the accuracy (ARMS) of another pulse oximetry was 2.7% [7] and better than another one showing poor validity [4].

Additionally, MultiSense^®^ demonstrated clinically acceptable accuracy in respiratory rate (RR) measurements. Both device placements showed higher performance and met accuracy standards, with an ARM of 2.52 rpm relative to the reference capnographic EtCO2 measurement. Given the acceptability threshold of ±5 rpm, these findings support the accuracy of MultiSense^®^ in RR monitoring.

A secondary objective was to compare measurement accuracy between chest and back placements for both respiratory rate and SpO_2_. Statistical analysis reveals no significant differences between the two positions, suggesting equivalent accuracy across placements. It has to be noted, however, that the Spo_2_ placement on the back exhibited a larger bias and, on the front, a higher variability. This bias might potentially be imputed to a lack of calibration of the SOC used, since another ISO80601-2-61 study comparing the device in the back placement against arterial blood oxygen in 10 subjects (clinical trials.gov NCT05466942) found no such bias of 0.3%. Our interpretation is that the increased variability on the chest placement might be imputed to a larger variability in the chest surface venous vasculature. The back placement is, therefore, preferred as it also offers increased quality of life for patients of both genders.

In terms of safety, adverse events were monitored and observed throughout the study. For adverse events attributable to the MultiSense device, only a few irritations and incidences of redness were observed, particularly at removal of the patch. All these were resolved after a few hours. Thus, there were no additional adverse events beyond the side effects anticipated in the protocol.

### 4.2. Limitations of the Study

The results presented in this report provide a good initial assessment of the measurement quality of the MultiSense device, particularly for SpO_2_. However, the results were generated on a relatively small number of patients (sixteen), which might limit the statistical power and generalizability of the findings. The entire 70–100% range could not be adequately represented. This could skew the perceived reliability of the device in severe hypoxia. Particularly, not all volunteers presented with a significant desaturation. In fact, the physiological responses to hypoxia involve a series of cardiorespiratory and tissue mechanisms that act to mitigate the reduction in tissue oxygen availability. Subjects presenting with an increased hypoxic ventilatory response might, therefore, reduce their decreases in SpO_2_ during a hypoxia test. Such physiological responses likely explain why some subjects remained with an SpO_2_ > 80%.

A proportion of patients, particularly those with a high phototype, did not show sufficient desaturation or at least below 80%. Additionally, most of the data recorded were above 85%, where accuracy is known to be the best. Consequently, the 70–85% range is partly under-represented. This restricts the conclusions that can be drawn regarding the devices’ performance across diverse populations, and measurements, therefore, need to be completed across the 70–100% SpO_2_ window.

Finally, we compared the MultiSense device with another pulse oximetry, which although representing a common standard, does not correspond exactly to the data obtained through arterial blood gas analysis. Although such indirect validation may not fully reflect all real-life clinical scenarios, oxygen saturation measured by non-invasive pulse oximetry is related to the arterial oxygen partial pressure (SpO_2_) by the sigmoidal oxygen–hemoglobin dissociation curve [31]. However, arterial blood gas is invasive and not deprived of potential complications, and comparing a new pulse oximeter device to a reference one is commonly used for research purposes before clinical application in real-life conditions.

## 5. Conclusions

In conclusion, the MultiSense device performed well in measuring SpO_2_ and suggested equivalence in the measurement accuracy between positioning on the back and positioning on the chest while remaining safe for users. MultiSense^®^ also maintained clinically acceptable accuracy in measuring respiratory rates, with the chest placement exhibiting a slightly higher performance compared to the back position.

Further studies investigating its value in patients with dark skin and strong desaturation in response to hypoxia would be useful before using MultiSense^®^ widely, both in healthy and diseased humans.

## Figures and Tables

**Table 1:** Bland–Altman differences, limits of agreement, and correlation coefficient for SpO_2_, with MultiSense^®^ in back and chest placements.

Placement	ARMS %	Difference (SoC–MultiSense^®^) % (μmol/min/g)	Limit of Agreements (Bias ± 1.96SD) (μmol/min/g)	Correlation (Pearson’s Coefficient) Between the Difference and the Average of the SpO_2_ Measurements (%) (μmol/min/g)
Back	2.94	1.65 [95% CI: 1.50, 1.74]	(−3.76, +7.05)	6.85
Chest	2.98	−0.12 [95% CI: −0.005, 0.265]	(−6.32, +6.08)	5.17

**Table 2:** Bland–Altman differences, limits of agreement, and correlation coefficient for SpO_2_ with MultiSense^®^ in back and chest placements.

Placement	ARMS %	Difference (SoC–MultiSense^®^) % (μmol/min/g)	Limit of Agreements (Bias ± 1.96SD) (μmol/min/g)	Correlation (Pearson’s Coefficient) Between the Difference and Average of the SpO_2_ Measurements (%) (μmol/min/g)
Back	2.52	−0.82 [95% CI: −1.15, −0.97	(−5.91, +4.26)	20.51
Chest	2.20	−0.50 [95% CI: −0.75, −0.55]	(−4.64, + 3.65)	3.02

## Data Availability

The data that support the findings of this study are available from the corresponding authors upon reasonable request.
